# Belzutifan‐Associated Reversible Heart Failure With Preserved Ejection Fraction in Metastatic Renal Cell Carcinoma

**DOI:** 10.1002/iju5.70266

**Published:** 2026-09-10

**Authors:** Hikaru Kimura, Shigeaki Nakazawa, Yu Ishizuya, Yoshiyuki Yamamoto, Kentaro Takezawa, Taigo Kato, Koji Hatano, Yoichi Kakuta, Atsunari Kawashima, Norio Nonomura

**Affiliations:** ^1^ Department of Urology, Graduate School of Medicine The University of Osaka Osaka Japan

**Keywords:** belzutifan, cardiotoxicity, heart failure with preserved ejection fraction, HIF‐2α inhibitor, metastatic renal cell carcinoma

## Abstract

**Background:**

Belzutifan, a hypoxia‐inducible factor 2α (HIF‐2α) inhibitor, is effective for metastatic renal cell carcinoma (mRCC), although its cardiovascular safety profile remains unclear.

**Case Presentation:**

A 52‐year‐old woman with mRCC developed progressive dyspnea and a 6.7‐kg weight gain 27 days after initiating belzutifan as third‐line therapy. Chest radiography revealed marked cardiomegaly (cardiothoracic ratio 63.4%), and echocardiography showed preserved left ventricular ejection fraction (67.1%) with mild diastolic dysfunction and findings consistent with congestive heart failure. Intensive diuretic therapy led to rapid improvement, with the cardiothoracic ratio decreasing to 42.7% and NT‐proBNP declining from 912 to 203 pg/mL. After belzutifan rechallenge at a reduced dose, NT‐proBNP increased again and subsequently normalized after discontinuation due to disease progression.

**Conclusion:**

Belzutifan may be associated with reversible congestive heart failure with preserved ejection fraction. Cardiac evaluation should be considered in patients who develop dyspnea during belzutifan therapy.

## Keynote Message

Although anemia and hypoxia are recognized adverse events of belzutifan, clinicians should be aware that congestive heart failure with preserved ejection fraction may also occur during treatment.

**Abbreviations**
CTR: cardiothoracic ratio
HFpEF: heart failure with preserved ejection fraction
HIF‐2α: hypoxia‐inducible factor 2α
ICI: immune checkpoint inhibitor
IMDC: International Metastatic Renal Cell Carcinoma Database Consortium
mRCC: metastatic renal cell carcinoma
NT‐proBNP: N‐terminal pro‐B‐type natriuretic peptide
RCC: renal cell carcinoma
TAPSE: tricuspid annular plane systolic excursion
TKI: tyrosine kinase inhibitor
VEGF: vascular endothelial growth factor
VHL: von Hippel–Lindau

## 1 Introduction

Belzutifan is a first‐in‐class hypoxia‐inducible factor 2α (HIF‐2α) inhibitor approved for the treatment of metastatic renal cell carcinoma (RCC) [1]. Clinical trials have identified anemia and hypoxia as its major adverse events, whereas clinically significant cardiac toxicity has not been reported in pivotal trials, pooled safety analyses, or pharmacovigilance studies [2, 3]. As the clinical use of belzutifan expands, recognition of rare adverse events is increasingly important. We report a case of reversible heart failure with preserved ejection fraction (HFpEF) that developed during belzutifan therapy and was supported by a positive dechallenge–rechallenge phenomenon.

## 2 Case Presentation

**FIGURE 1:**
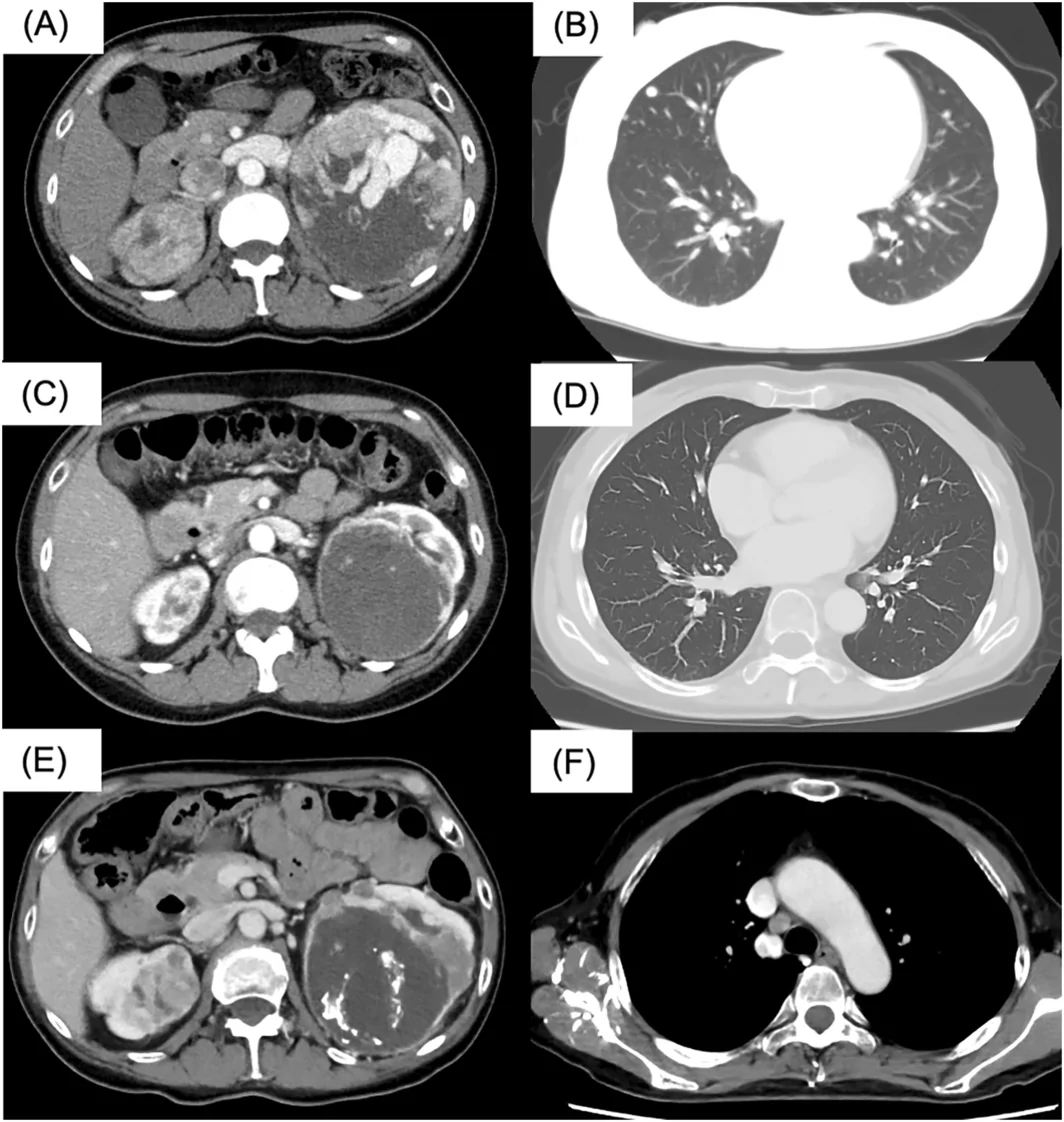
Serial contrast‐enhanced computed tomography findings during systemic therapy. (A, B) At initial diagnosis, contrast‐enhanced computed tomography demonstrated bilateral renal tumors (A) and pulmonary metastases (B). Pancreatic metastasis was also identified but is not shown. The disease was staged as cT3aN0M1. (C, D) After prior systemic therapy, the pulmonary and pancreatic metastases had achieved complete response, whereas the bilateral renal tumors remained. (E, F) Immediately before initiation of belzutifan, the bilateral renal tumors showed disease progression (E), and new mediastinal and right hilar lymph node metastases were identified (F), leading to initiation of belzutifan as third‐line therapy.

A 52‐year‐old woman with a history of hypertension and hypothyroidism was diagnosed with clear cell renal cell carcinoma by CT‐guided percutaneous renal biopsy. Initial imaging demonstrated bilateral renal tumors with pulmonary and pancreatic metastases, and the disease was staged as cT3aN0M1 and classified as intermediate risk according to the International Metastatic Renal Cell Carcinoma Database Consortium criteria (Figure 1A,B). After disease control for 49 months with first‐line avelumab plus axitinib and 47 months with second‐line cabozantinib, pulmonary and pancreatic metastases achieved and maintained complete response (Figure 1C,D). However, bilateral renal tumors progressed and new mediastinal and right hilar lymph node metastases developed, leading to initiation of belzutifan (120 mg once daily) as third‐line therapy (Figure 1E,F). At the initiation of belzutifan, the hemoglobin level was 11.0 g/dL, estimated glomerular filtration rate (eGFR) was 68.5 mL/min/1.73 m [2], and urinalysis was negative for proteinuria.

**FIGURE 2:**
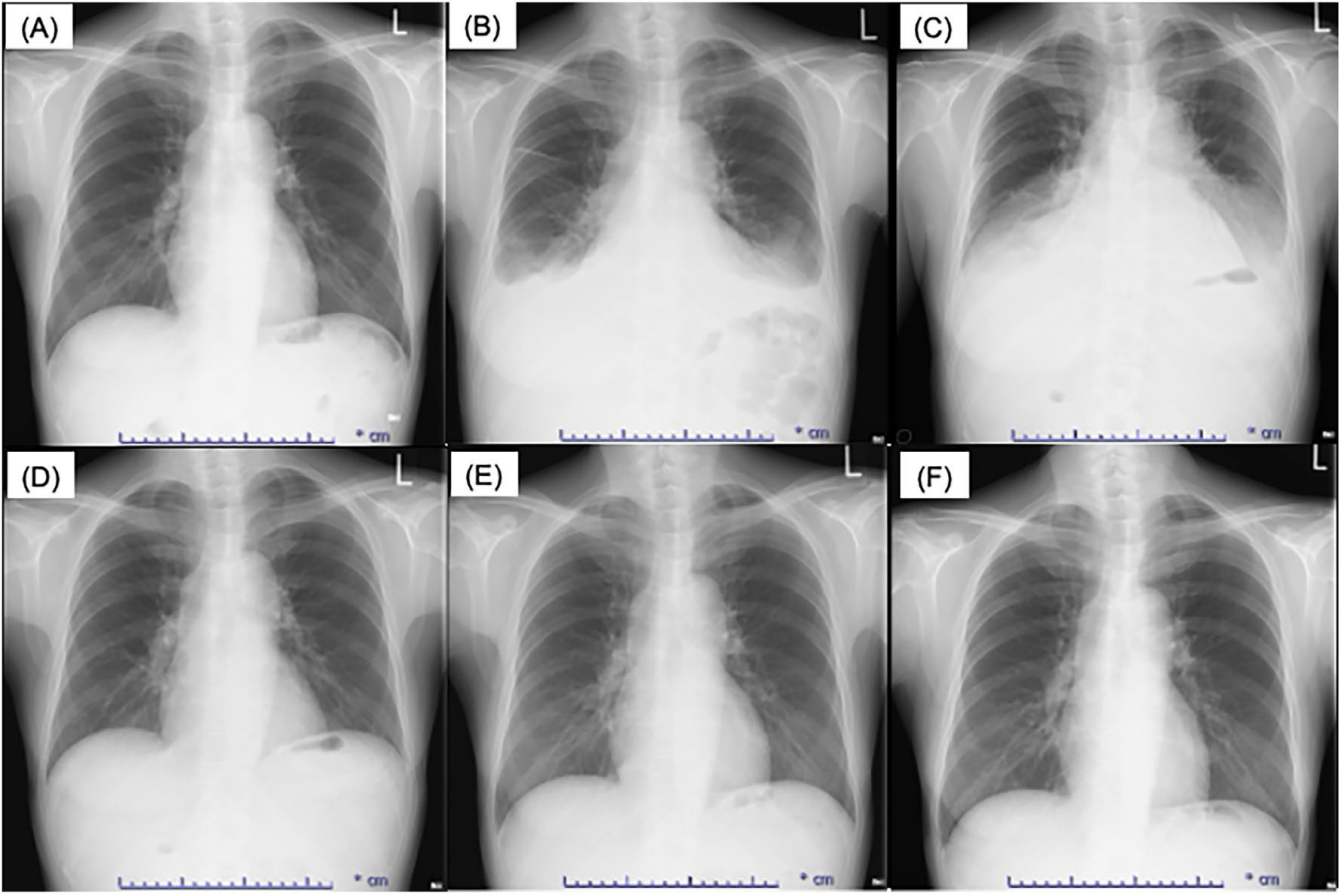
Serial chest radiographs during the clinical course. (A) At initiation of belzutifan therapy, showing normal cardiac size (CTR, 41.4%) without pleural effusion. (B) On the day of admission (27 days after belzutifan initiation), demonstrating marked cardiomegaly (CTR, 63.4%) and bilateral pleural effusions. (C) Hospital day 5, showing transient worsening of pulmonary congestion and pleural effusion despite initiation of heart failure therapy. (D) Hospital day 16, on the day of belzutifan rechallenge, demonstrating improvement in cardiomegaly (CTR, 47.3%). (E) Eighteen days after belzutifan rechallenge, showing no recurrence of cardiomegaly (CTR, 44.8%). (F) One day after discontinuation of belzutifan, showing normal cardiac size (CTR, 42.1%).

Twenty‐seven days after starting belzutifan, she presented with rapidly progressive dyspnea and bilateral lower‐extremity edema. Her body weight had increased from 53.9 to 60.6 kg. Oxygen saturation was 91% on room air. Laboratory testing demonstrated a hemoglobin level of 8.9 g/dL, an eGFR of 64.5 mL/min/1.73 m [2], proteinuria of 0.28 g/gCr, and an elevated NT‐proBNP level of 912 pg/mL. Chest radiography at the initiation of belzutifan showed no cardiomegaly or pleural effusion (Figure 2A). At presentation 27 days later, chest radiography demonstrated marked cardiomegaly (cardiothoracic ratio [CTR], 63.4%) and bilateral pleural effusions (Figure 2B).

Transthoracic echocardiography revealed preserved left ventricular ejection fraction (67.1%) with mild diastolic dysfunction. These findings were consistent with heart failure with preserved ejection fraction (HFpEF), corresponding to CTCAE version 5.0 Grade 3 heart failure (Table 1).

**TABLE 1:** Comparison of baseline echocardiographic findings and those at the onset of heart failure.

Parameter	Baseline echocardiography	At heart failure onset
LVEF (%)	72	67.1
LVDd (mm)	48	54.5
LVDs (mm)	28	34
E/A ratio	0.74	0.7
DcT (ms)	187	197
LAD (mm)	42	39
E/e’	8.1	8.6
LAVI	39.9	Not assessable
IVC (mm)	17/6	26/22

*Note:* Baseline echocardiography, performed at initiation of first‐line avelumab plus axitinib therapy, demonstrated preserved left ventricular systolic function with mild diastolic dysfunction. At the onset of heart failure, left ventricular and inferior vena cava dimensions increased, whereas left ventricular systolic function remained preserved.

Abbreviations: DcT, deceleration time; E/e′, ratio of early mitral inflow velocity to early diastolic mitral annular velocity; IVC, inferior vena cava; LAD, left atrial diameter; LAVI, left atrial volume index; LVDd, left ventricular end‐diastolic diameter; LVDs, left ventricular end‐systolic diameter; LVEF, left ventricular ejection fraction.

**FIGURE 3:**
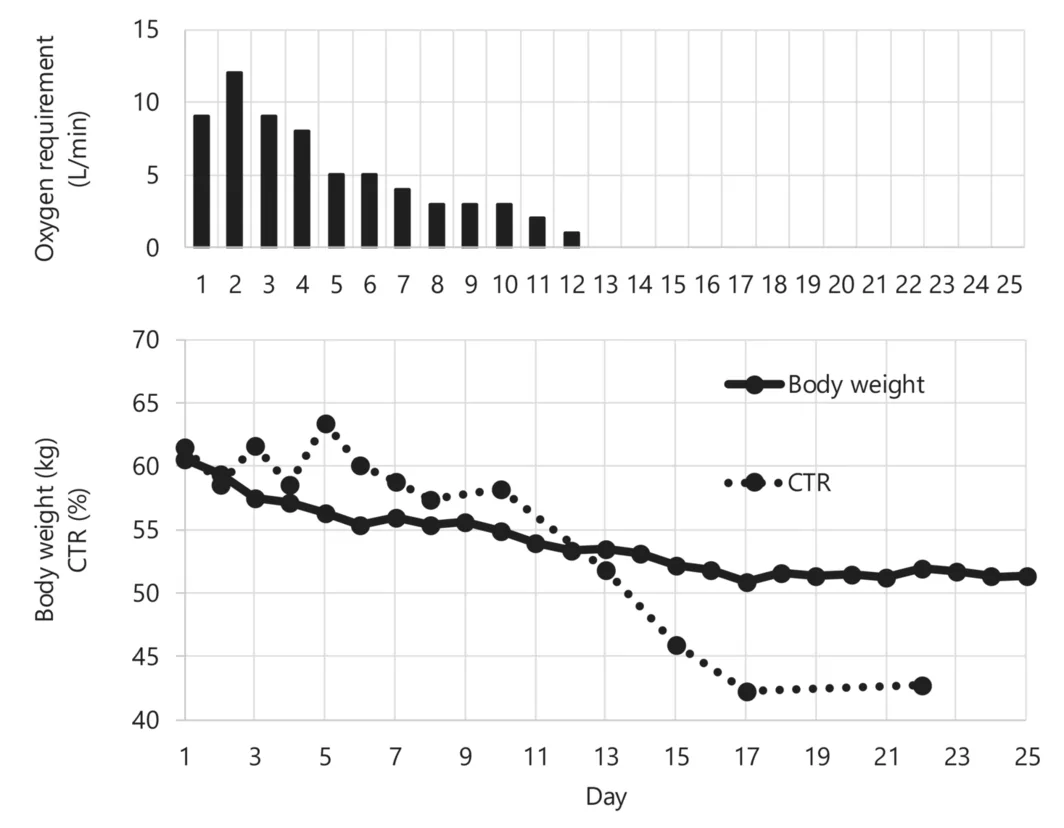
Clinical course after hospitalization. Changes in body weight (solid circles), cardiothoracic ratio (CTR; squares), and oxygen requirement (triangles) during hospitalization are shown. Belzutifan was discontinued on admission, followed by initiation of heart failure therapy, resulting in progressive reductions in body weight, CTR, and oxygen requirement. Belzutifan rechallenge at a reduced dose (80 mg) was performed on hospital day 16.

Belzutifan was discontinued on the day after admission, and treatment with candesartan, azosemide, spironolactone, and intravenous furosemide was initiated. Her respiratory condition temporarily worsened, requiring up to 12 L/min of supplemental oxygen via reservoir mask (Figure 2C). However, she improved rapidly with diuretic therapy. By hospital day 13, oxygen supplementation was discontinued. NT‐proBNP decreased to 203 pg/mL, CTR improved to 42.7%, and body weight decreased to 51.9 kg (Figures 2D, 3).

On hospital day 16, belzutifan was resumed at a reduced dose of 80 mg daily. Eighteen days later, NT‐proBNP increased from 203 to 482 pg/mL despite the absence of overt heart failure symptoms. Chest radiography showed no recurrence of cardiomegaly (CTR, 44.8%; Figure 2E). Belzutifan was subsequently discontinued because of radiographic disease progression. Following discontinuation, NT‐proBNP gradually normalized and chest radiography showed resolution of cardiomegaly (Figure 2F, Figure 3).

## 3 Discussion

Belzutifan has demonstrated clinical efficacy in advanced renal cell carcinoma and is generally associated with manageable adverse events, primarily anemia and hypoxia. Importantly, heart failure or clinically significant cardiac dysfunction was not reported as a treatment‐related adverse event in the Phase 3 LITESPARK‐005 trial [2]. Similarly, pooled safety analyses [2] and a recent pharmacovigilance study based on the FDA Adverse Event Reporting System [3] did not identify heart failure as a significant safety signal. In addition, FDA multidisciplinary review documents did not highlight cardiac dysfunction as a recognized risk. Taken together, these findings suggest that clinically apparent heart failure associated with belzutifan is exceedingly rare and may be underrecognized in routine clinical practice.

Baseline echocardiography performed at initiation of first‐line avelumab plus axitinib therapy demonstrated preserved left ventricular systolic function with mild diastolic dysfunction but no clinically overt heart failure. As the patient remained free of cardiac symptoms or signs of heart failure during subsequent treatment, repeat echocardiography was not performed until the onset of heart failure shortly after belzutifan initiation. Although the available echocardiographic findings do not allow a definitive diagnosis of intrinsic HFpEF because several diastolic parameters could not be assessed during the acute phase, the combination of preserved ejection fraction, marked cardiomegaly, bilateral pleural effusions, inferior vena cava dilatation, elevated NT‐proBNP, and rapid response to diuretic therapy supported acute congestive heart failure with preserved ejection fraction. Belzutifan‐related fluid retention may have contributed to the development of volume overload in this patient.

Delayed cardiotoxicity associated with immune checkpoint inhibitors should also be considered. Villatore et al. reported biopsy‐confirmed myocarditis during belzutifan plus lenvatinib therapy and considered delayed pembrolizumab‐associated cardiotoxicity to be the most likely cause [4], although a contribution from belzutifan or lenvatinib could not be excluded. In our patient, cabozantinib was discontinued the day before initiation of belzutifan. No episodes of heart failure, cardiomegaly, or clinically apparent cardiac dysfunction had occurred during the preceding 49 months of avelumab plus axitinib therapy or the subsequent 47 months of cabozantinib therapy. Furthermore, the temporal relationship between belzutifan initiation, prompt improvement after withdrawal, and re‐elevation of NT‐proBNP after rechallenge supports belzutifan as the more likely precipitating factor, although a contributory effect of previous systemic therapy cannot be completely excluded.

The mechanism underlying belzutifan‐associated cardiac decompensation remains uncertain. Peripheral edema and weight gain have been reported during belzutifan therapy, whereas clinically overt heart failure has not been recognized as a characteristic adverse event. Therefore, belzutifan‐related fluid retention may have contributed to the marked congestion observed in this patient. However, the substantial elevation in NT‐proBNP, marked cardiomegaly, bilateral pleural effusions, inferior vena cava dilatation, and rapid response to heart failure therapy suggest that this presentation represented clinically significant cardiac congestion rather than isolated peripheral edema. HFpEF is characterized by impaired ventricular relaxation and increased ventricular stiffness, which can lead to elevated filling pressures and congestion despite preserved systolic function [5]. HIF‐2α plays an important role in cellular adaptation to hypoxia and metabolic homeostasis, and its inhibition may impair cardiovascular adaptation in susceptible individuals [6]. Further studies are required to clarify the mechanisms underlying this rare complication.

In conclusion, this case suggests that belzutifan may be associated with reversible congestive heart failure with preserved ejection fraction. Clinicians should consider cardiac evaluation when patients receiving belzutifan develop dyspnea, weight gain, or hypoxemia rather than attributing these symptoms solely to belzutifan‐related hypoxia or anemia.

## Consent

Written informed consent was obtained from the patient for the publication of this case report and the accompanying images.

## Conflicts of Interest

Kentaro Takezawa is an Editorial Board member of International Journal of Urology and a co‐author of this article. To minimize bias, he was excluded from all editorial decision‐making related to the acceptance of this article for publication.

## Data Availability

The data that support the findings of this study are available on request from the corresponding author. The data are not publicly available due to privacy or ethical restrictions.
